# Spatial transcriptomic differences in the breast muscle of grower broilers at 21 and 28 days of age

**DOI:** 10.1016/j.psj.2025.105095

**Published:** 2025-03-24

**Authors:** Janghan Choi, Jihwan Lee, Doyun Goo, Gippeum Han, Venkata Sesha Reddy Choppa, Seshidhar Reddy Gudidoddi, Majid Shakeri, Hong Zhuang, Brian Bowker, Woo Kyun Kim, Byungwhi Kong

**Affiliations:** a Department of Animal and Food Sciences, Texas Tech University, Lubbock, TX 79409, USA; b Department of Poultry Science, University of Georgia, Athens, GA 30602, USA; c US National Poultry Research Center, USDA-ARS, Athens, GA 30605, USA

**Keywords:** Pectoralis major, Caudal area, Cranial area, Broiler, Transcriptomics

## Abstract

This study investigated transcriptomic differences between the caudal and cranial areas in breast muscle in the grower phase of broiler chickens (D 21 and D 28). A total of 66 one-day-old broiler chickens were allotted to 6 cage pens of 11 birds per pen. On D 21 and D 28, one bird per pen was randomly selected, and breast muscle samples were collected in the caudal and cranial areas of the *Pectoralis major*. RNA sequencing was conducted, followed by screening for differentially expressed genes (DEGs; *P* < 0.05) and multivariate analyses. A total of 24,498 genes were identified, and 8,831 genes had greater than 100 mean read count. On D 21, there were 666 DEGs, among which 482 and 184 genes were down-regulated and up-regulated, respectively, in the caudal area compared with the cranial area. There were 2 down-regulated and 56 up-regulated genes with a greater than 1.5-fold change (FC). On D 28, there were 872 DEGs, among which 408 and 464 genes were down-regulated and up-regulated, respectively. There were 12 down-regulated and 23 up-regulated genes with a greater than 1.5-fold change. Principal component analysis (PCA) plots showed that gene profiles were not distinctly separated between the caudal area and the cranial area of breast muscle on D 21 and D 28. On D 21, collagen type XI alpha 1 chain (COL11A1), fibromodulin (FMOD), myosin heavy chain 7B (MYH7B), carbonic anhydrase 3A (CA3A), tenomodulin (TNMD), COL12A1, carboxypeptidase Z (CPZ), lysyl oxidase like 2 (LOXL2), COL1A1, and COL1A2 were significantly down-regulated in the caudal area compared to the cranial area with a greater than 2-FC (*P* < 0.05). KEGG pathway enrichment analysis indicated that various pathways including regulation of actin cytoskeleton, ribosome, and focal adhesion were significantly different in the caudal area compared with the cranial area. These findings suggest that spatial differences in gene expression within the breast muscle could be linked to functional or developmental variations between regions, potentially influencing muscle growth, meat quality, and breast myopathies. Understanding these spatial transcriptomic differences may provide insights into identifying etiologies and developing strategies for breast myopathies.

## Introduction

Effective genetic selection and nutritional programs have dramatically improved the productivity and efficiency of broiler production, making broiler meat one of the most nutritious and affordable animal protein sources (Choi et al., 2023). Around 45 kg of broiler meat per capita was consumed in the US in 2022, making it the most consumed meat in the US (Shahbandeh, 2023). Modern broiler chickens have 3.7 to 4.7 times greater body weight and more than double the size of breast muscles compared to traditional lines (The Athens Canadian Random Bred; ACRB) (Collins et al., 2014).

It is noteworthy that muscle myopathies including white stripping (WS), wooden breast (WB), and spaghetti meat (SM), which negatively affect meat quality (Choi et al., 2024; Tasoniero et al., 2022), are frequently observed in the breast meat of fast-growing broiler chickens (Che et al., 2022a). While the incidence of breast myopathies varies across studies, it can reach up to 90 % in modern broilers with most fillets displaying multiple myopathies (Che et al., 2022b; Kuttappan et al., 2016). These conditions are estimated to result in annual economic losses of $200 million to $1 billion for the U.S. poultry industry (Barbut, 2025; Kuttappan et al., 2016). Although the symptoms of breast myopathies are mainly observed in the finisher phase, these conditions are known to initiate at a young age (around D 14 to 21) in broiler chickens (Papah et al., 2018). Muscle growth in broiler chickens also accelerates in the grower phase (D 21 to D 28) (Scheuermann et al., 2003). The symptoms of these breast myopathies predominantly manifest in the cranial area rather than the caudal area of the *Pectoralis major* muscle (Baldi et al., 2018; Kong et al., 2024; Zhang et al., 2023).

Variations in the development of muscle structures, connective tissue, protein synthesis, and other factors may exist between the caudal and cranial areas of the breast muscle (Clark and Velleman, 2016). These differences could potentially explain the higher occurrence of muscle myopathies in the cranial area. However, limited research has been conducted to directly compare the caudal and cranial regions of the breast muscle. Investigating these spatial transcriptomic differences may provide deeper insights into both regional differences in biological pathways in breast muscle and identifying etiologies and developing strategies for breast myopathies. Furthermore, understanding the differences between the caudal and cranial areas of chicken breast muscle during the grower phase is important, since cranial sides grow faster and become thicker than caudal sides, and breast myopathies may onset during this grower period (Brothers et al., 2019). Hence, the purpose of the study was to investigate transcriptomic differences between the caudal and cranial areas in chicken breast muscle during the grower phases (D 21 and D 28).

## Materials and methods

### Animals, experimental design, and sampling

**Fig. 1:**
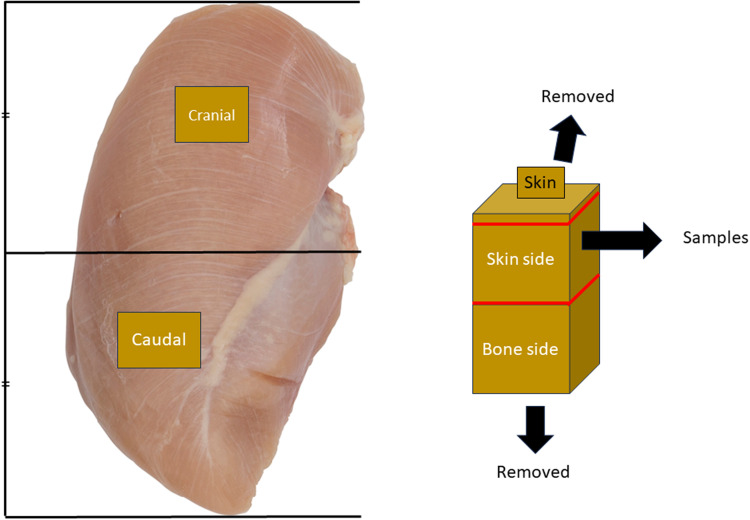
Schematic illustration of sampling areas. The caudal and cranial areas were identified by separating the chicken breast muscle in half. The mid-area of the caudal and cranial portions were collected, and the skin side of samples were collected after removing bone side and skin of chicken breast muscle.

The Institutional Animal Care and Use Committee of the University of Georgia, Athens, GA approved the animal use protocol for the current study (A2021 12-012). A total of 66 one-day-old male Cobb500 broiler chickens were allotted to 6 cage pens of 11 birds per pen. Diets were formulated to meet and exceed the requirements of the nutrition requirement guide of CobbVantress (2022). On D 21 and D 28, one bird per pen (*N* = 6 per group) was randomly selected, and breast muscle samples were collected from the caudal and cranial portions of the *Pectoralis major* as shown in Fig. 1. Approximately 2 cm × 2 cm × 2 cm samples were collected from the caudal and cranial areas just below the skin-side surface of the muscle. Samples were snap-frozen in liquid nitrogen and stored at −80°C for further analysis.

### RNA extraction and library preparation

RNA was extracted from the frozen muscle samples as described by Khatri et al. (2018) and Kong et al. (2017). Briefly, 100 mg of tissue were homogenized using a beadblaster (Benchmark Scientific, Edison, NJ) and total RNA was extracted using 1 mL Trizol (ThermoFisher, Waltham, MA) following manufacturer instructions. RNA precipitation and DNase digestion were conducted using Qiagen's RNeasy spin columns (Qiagen, Valencia, CA) according to manufacturer instructions. The quality and quantity of extracted RNA samples were determined using Agilent 4200 Tapestation (Agilent, Santa Clara, CA) and NanoDrop One (ThermoFisher, Waltham, MA). Total RNA in up 20 μL with RNA integrity number (RIN) score higher than 9 was subjected to the downstream library preparation. RNA sequencing was conducted at SeqMatic LLC (Fremont, CA). Briefly, library preparation was performed by using Illumina Stranded mRNA ligation kit and Illumina RNA UD Adapters (Illumina, San Diego, CA). The library was sequenced on the Illumina Novaseq X Plus instrument (Illumina) using a 150 bp paired-end read method. Each sample was sequenced to a minimum of 20 million reads per sample. Raw reads were filtered by removing adapters, poly-N, and low quality reads. A reference index of GRC7b Gallus gallus genome and annotation downloaded from ENSEMBL was built using HISAT2 v2.2.1 (Zhang et al., 2021). Differential expression comparing caudal- with cranial parts was conducted using DEseq2 module in R software.

### Data processing and multivariate statistical analyses

One bird per pen was considered as an experimental unit, and there were 6 replicates per group. For all analyses, genes showing mean read count values (average normalized read counts in both groups) under 100, which indicate very low expression, were removed. Volcano plots were constructed using GraphPad Prism software version 9.1.0 (GraphPad Software Inc., San Diego, CA) with two conditions [(*P* < 0.05 and |log_2_fold change (FC)| > 0.5849 (1.5-FC)]. MA plots were constructed using mean read count and FC. Principal component analysis (PCA) was performed using base package of R software (version 4.1.2) with parameter scale=True indicating unit variance scaling (UV) for normalizing the data.

Differentially down- and up-regulated genes with a greater than 2-FC (|log_2_FC|>1) were screened. Consistently, differentially expressed genes (DEGs) on D 21 and D 28 were selected by screening DEGs (*P* < 0.05) in the same direction (either up-regulated or down-regulated). The DEGs (*P* < 0.05) were subjected to gene ontology (GO) and the KEGG pathway enrichment analysis by using WebGestalt (WEB-based Gene SeT AnaLysis Toolkit) (Kanehisa and Goto, 2000).

## Results and discussion

### Number of differentially expressed genes (DEGs) and multivariate analyses

**Fig. 2:**
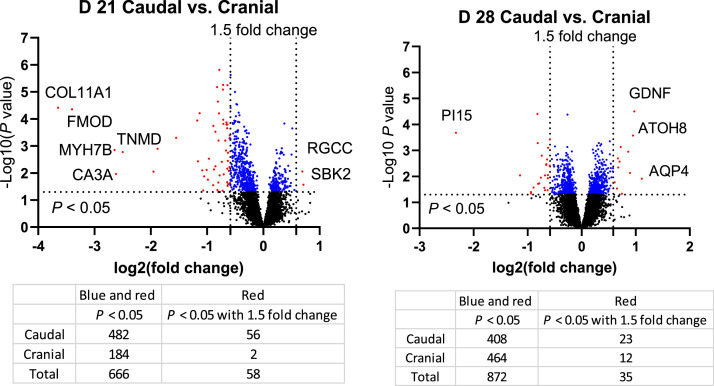
Volcano plots of differentially expressed genes (DEGs) in the caudal area compared with the cranial area of chicken breast muscle on D 21 and D 28. Black dots indicate genes with *P* > 0.05. Blue and red dots indicate DEGs (*P* < 0.05). Red dots indicate DEGs (*P* < 0.05) with a greater than 1.5-fold change. Notable DEGs include COL11A1 (Collagen type XI alpha 1 chain), FMOD (Fibromodulin), MYH7B (Myosin heavy chain 7B), TNMD (Tenomodulin), CA3A (Carbonic anhydrase 3A), RGCC (Regulator of cell cycle), SBK2 (SH3 domain binding kinase family member 2), PI15 (Peptidase inhibitor 15), GDNF (Glial cell line-derived neurotrophic factor), ATOH8 (Atonal BHLH transcription factor 8), and AQP4 (Aquaporin 4). Sample number: *N* = 6.

**Fig. 3:**
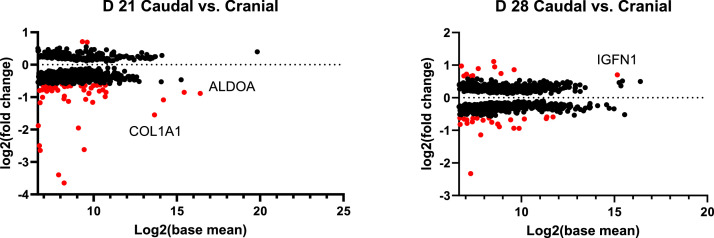
MA plots of differentially expressed genes (DEGs) (*P* < 0.05) with mean read counts in the caudal area compared with the cranial area of chicken breast muscle on D 21 and D 28. Red dots indicate DEGs (*P* < 0.05) with a greater than 1.5-fold change. Notable DEGs include ALDOA (Aldolase, fructose-bisphosphate A), COL1A2 (Collagen type I alpha 2 chain), and IGFN1 (Immunoglobulin-like and fibronectin type III domain containing 1). Sample number: *N* = 6.

**Fig. 4:**
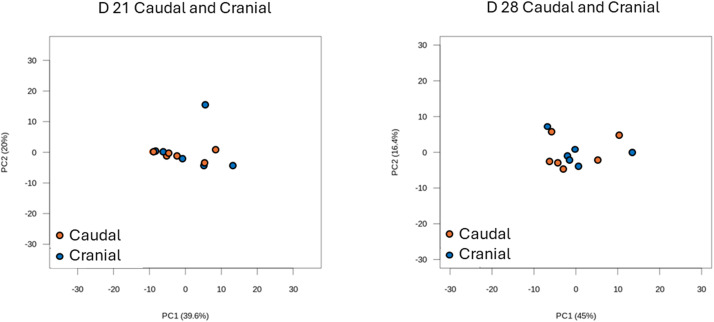
Principal component analysis (PCA) in the caudal area compared with the cranial area of chicken breast muscle on D 21 and D 28. Sample number: *N* = 6.

On D 21 and D 28, a total of 24,498 genes were identified and 8,831 genes had a mean read count greater than 100. As shown in Fig. 2, on D 21, there were 666 DEGs, including 482 and 184 genes down- and up-regulated, respectively, in the caudal area compared with the cranial area. Of those, 56 up- and 2 down-regulated DEGs showed a greater than 1.5-FC. On D 28, there were 872 DEGs, 408 and 464 genes were down-regulated and up-regulated, respectively, in the caudal area compared with the cranial area. There were 23 up-regulated and 12 down-regulated DEGs with a greater than 1.5-FC in the caudal area compared with the cranial area. The MA plot showed the DEGs with values of log_2_FC and levels of expression (mRNA abundance) (Fig. 3). The PCA plots showed that gene profiles were not distinctly separated in the caudal area compared with the cranial area of breast muscle on D 21 and D 28 (Fig. 4).

These results suggest that genes were mostly down-regulated in the caudal area compared to the cranial area. Brothers et al. (2019), who compared gene expression profiles in the cranial and caudal areas of chicken breast muscle at 21-day-old, showed that 8 and 4 genes were down- and up-regulated in the cranial area compared to caudal area, respectively. The reason for a greater number of DEGs in the current study compared to the previous study by Brothers et al. (2019) may be attributed to the use of non-adjusted *P* values. Non-adjusted *P* values were used in the current study since it was aimed at gaining a more comprehensive picture with a greater number of genes with a less stringent cut off level. Despite the substantial number of DEGs identified, the gene expression profiles of the caudal and cranial regions remained largely similar.

### Differentially expressed genes with a greater than 2-fold change

DEGs with a greater than 2-FC in the caudal area compared with the cranial area of breast muscle on D 21 and D 28 are presented in Table 1. There were more DEGs with 2-FC on D 21 (15) compared to D 28 (3). Except aquaporin 4 (AQP4) on D 28, all genes, including collagen type XI alpha 1 chain (COL11A1), fibromodulin (FMOD), myosin heavy chain 7B (MYH7B), carbonic anhydrase 3A (CA3A), tenomodulin (TNMD), COL12A1, carboxypeptidase Z (CPZ), lysyl oxidase like 2 (LOXL2), COL1A1, COL1A2, and tropomyosin 2 (TPM2) on D 21 and PI (peptidase inhibitor 15) on D 28, were down-regulated in the caudal area compared to the cranial area with a greater than 2-FC (*P* < 0.05).

**Table 1:** Differentially expressed genes (DEGs; *P* < 0.05) with a greater than 2-fold change in the caudal area compared with the cranial area in chicken breast muscle on D 21 and D 281.

Gene name	Mean read count	Log_2_(fold change)	*P* value
D 21			
COL11A1	299.21	−3.646	0.00004
FMOD	238.06	−3.398	0.00004
MYH7B	110.70	−2.644	0.00142
CA3A	690.08	−2.619	0.01091
TNMD	106.52	−2.499	0.00167
COL12A1	541.50	−1.951	0.00893
CPZ	101.93	−1.879	0.00127
COL1A1	12843.68	−1.550	0.00050
SEMA3F	109.83	−1.173	0.00012
ENSGALG00010026410	982.63	−1.161	0.00373
LOXL2	358.33	−1.131	0.00006
COL1A2	18697.88	−1.084	0.00795
TPM2	731.86	−1.067	0.04391
ENSGALG00010000589	297.27	−1.051	0.01325
ENSGALG00010004092	121.85	−1.006	0.00770
D 28			
PI15	154.26	−2.325	0.00021
ENSGALG00010002788	225.09	−1.141	0.00915
AQP4	364.52	1.112	0.01219

1 Sample number: *N* = 6; COL11A1: collagen type XI alpha 1 chain; FMOD: fibromodulin; MYH7B: myosin, heavy chain 7B; CA3A: carbonic anhydrase 3A; TNMD: tenomodulin; COL12A1: collagen type XII alpha 1 chain; CPZ: carboxypeptidase Z; COL1A1: collagen type I alpha 1 chain; SEMA3F: semaphorin 3F; LOXL2: lysyl oxidase like 2; COL1A2: collagen type I alpha 2 chain; TPM2: tropomyosin 2; PI15: peptidase inhibitor 15; AQP4: aquaporin 4.

Decreased expression of MYH7B, CPZ, and TPM2 in the caudal area may indicate that there could be differences in muscle synthesis and contraction between the caudal and cranial areas. Myosin heavy chain is the motor protein of muscle thick filaments (Wells et al., 1996). CPZ regulates the development of skeletal elements in the chicken (Moeller et al., 2003). Tropomyosin regulates muscle contraction by interacting with diverse actin binding proteins (El-Mezgueldi, 2014). These results indicated cranial area may retain both altered contraction patterns and a greater number of muscle fibers compared to the caudal area. With respect to incidence of breast myopathies, Puolanne et al. (2021) demonstrated that WB meat had significantly greater tensile strength in the cranial area compared to the medial area. Nonetheless, increased expression of genes related to muscle contraction could indicate altered contraction patterns with denser muscle fibers in the cranial area, which could also influence rigor mortis development postmortem.

COL11A1, COL12A1, COL1A1, COL1A2, FMOD, TNMD, and LOXL2 are closely related to the functionality and formation of connective tissue, which have an important role in supporting muscle fibers. COL1A1 and COL1A2 are the most abundant collagen types in the breast muscle and have an important role in maintaining tissue integrity (Nishimura et al., 2024; Sand et al., 2016). In the current study, the mRNA abundances of COL1A1 and COL1A2 were notably high with values of 12,843.68 and 18,697.88, respectively. In contrast, Brothers et al. (2019) showed that COL11A1, COL12A1, and COL22A1 were down-regulated in the cranial area compared to the caudal area in breast muscle. FMOD, found predominantly in articular cartilage, binds to collagen and affects collagen fibrillogenesis and cross-linking (Kalamajski et al., 2016). Lower expression of FMOD in the caudal area could be associated with lower expression of genes related to collagen (Zhao et al., 2023). Tenomodulin (TNMD) is a well-known maturity marker for tendon and ligament lineage cells (Lin et al., 2017). These results suggested that there could be more connective tissue in the cranial area compared to the caudal area of chicken breast muscle due to the cranial area having more contact area with the keel bone and wings and a greater density of muscle fibers (Smith and Fletcher, 1988), which could require more connective tissue for structural support. Differences in connective tissue can affect muscle development, function, and meat quality (Velleman, 2020). Furthermore, overproduction of collagens is one of the symptoms of meat with breast myopathies (WB and SM) (Bian et al., 2024; Zhu et al., 2023). A recent study by Li et al. (2025) demonstrated that the WB had greater expression of COL1A1 compared to the normal chicken breast meat. The cranial area showed greater expression of major collagen types, suggesting that this region may be more susceptible to developing breast myopathies compared to the caudal area.

Greater expression of COL11A1 and COL12A1 have been associated with diverse diseases including muscle diseases (e.g., myopathies) and cancers (Jiang et al., 2019; Nallanthighal et al., 2021; Wu et al., 2014; Zou et al., 2014). Potentially, a greater expression of COL11A1 and COL12A1 may indicate that the cranial area of chicken breast muscle would be vulnerable to breast myopathies such as WB and SM in broiler chickens. Zhang et al. (2023) demonstrated that the characteristic symptoms of WB (e.g., hard texture) are most severe in the cranial area of the muscle. The symptoms of SM are also mainly shown in the cranial area of the breast muscle in broiler chickens (Baldi et al., 2018; Che et al., 2022a). Vascular health and oxidative stress are considered as potential etiologies for breast myopathies in broiler chickens (Ayansola et al., 2021). Greater expression of LOXL2 and CA3A in the cranial portion of the muscle suggest that differences in vascular health and oxidative stress (Räisänen et al., 1999; Steppan et al., 2019) may make this portion of the muscle more susceptible to myopathy development than the caudal portion. These results further indicate that the cranial area contains a greater number of muscle fibers and could be more vulnerable to breast myopathy development in broiler chickens.

### Top 5 down- and up-regulated genes and consistently regulated genes on D 21 and D 28

The top 5 up- and down-regulated genes in the caudal area compared with the cranial area of breast muscle are shown in Table 2. On D 28, peptidase inhibitor 15 (PI15), elastin (ELN), and myosin heavy chain 11 (MHC11) were in the top 5 down-regulated genes in the caudal area compared with the cranial area. PI15 has an important role in regulating collagen synthesis (Galant et al., 2019) and elastin is a component of connective tissue (Wang et al., 2021). Myosin heavy chain is the motor protein of muscle thick filaments (Wells et al., 1996). The downregulation of PI15 and ELN in the caudal region may suggest reduced collagen regulation and impaired connective tissue development aligning with the observed decrease in connective tissues in the caudal region in the present study.

**Table 2:** The 5 highest log_2_(fold change) values for down- and up-regulated differentially expressed genes (DEGs; *P* < 0.05) in the caudal area compared with the cranial area in chicken breast muscle on D 21 and D 281.

Gene name	Mean read count	Log_2_(fold change)	*P* value
D 21			
Top 5 down-regulated			
COL11A1	299.2	−3.646	0.00004
FMOD	238.1	−3.398	0.00004
MYH7B	110.7	−2.644	0.00142
CA3A	690.1	−2.619	0.01091
TNMD	106.5	−2.499	0.00167
Top 5 up-regulated			
SBK2	639.2	0.711	0.02706
RGCC	779.5	0.693	0.00879
UCHL1	749.3	0.551	0.04250
GAS6	247.5	0.517	0.00022
GGCT	100.5	0.501	0.00209
D 28			
Top 5 down-regulated			
PI15	154.3	−2.325	0.00021
ENSGALG00010002788	225.1	−1.141	0.00915
ELN	938.3	−0.938	0.04011
ENSGALG00010022968	773.5	−0.937	0.04529
MYH11	451.2	−0.890	0.02720
Top 5 up-regulated			
AQP4	364.5	1.112	0.01219
GDNF	109.3	0.974	0.00003
ATOH8	377.7	0.947	0.00027
COL19A1	205.0	0.896	0.00733
RGCC	779.5	0.862	0.00111

1 Sample number: *N* = 6; COL11A1: collagen type XI alpha 1 chain; FMOD: fibromodulin; MYH7B: myosin, heavy chain 7B; CA3A: carbonic anhydrase; TNMD: tenomodulin; SBK2: SH3 domain binding kinase family member 2; RGCC: regulator of cell cycle; UCHL1: ubiquitin C-terminal hydrolase L1; GAS6: growth arrest specific 6; GGCT: gamma-glutamylcyclotransferase;; PI15: peptidase inhibitor 15; ELN: elastin; MYH11: myosin, heavy chain 11; AQP4: aquaporin 4; GDNF: glial cell derived neurotrophic factor; ATOH8: atonal bHLH transcription factor 8; COL19A1: collagen type XIX alpha 1 chain; RGCC: regulator of cell cycle.

In both D 21 and D 28 samples, fifteen genes showed consistently altered expression patterns (*P* < 0.05 and same direction) in the caudal area compared with the cranial area of the breast muscle (Table 3**)**. However, this was considered a low number because there were 666 and 872 DEGs in the comparison on D 21 and D 28, respectively. These results also suggest that the differences between the caudal and cranial areas could be derived from differential age-dependent development.

**Table 3:** Consistently altered (*P* < 0.05 and same direction) differentially expressed genes (DEGs) on D 21 and D 28 in the caudal area compared with the cranial area in chicken breast muscle1.

		D 21		D 28	
	Mean read count	Log_2_(fold change)	*P* value	Log_2_(fold change)	*P* value
OAZ1	7570.7	0.373	0.0001	0.961	0.0396
GPC2	2358.1	0.394	0.0079	0.552	0.0004
FDPS	1044.2	0.470	0.0085	0.989	0.0439
RGCC	779.5	0.693	0.0088	0.587	0.0011
PARP3	133.8	0.391	0.0183	1.010	0.0470
ENSGALG00010015356	133.2	0.238	0.0190	0.981	0.0426
HSPB2	579.0	0.409	0.0208	0.747	0.0114
ENSGALG00010030072	1476.6	0.328	0.0216	0.721	0.0089
DHCR24	333.6	0.490	0.0234	0.743	0.0110
LOC422270 LOC422270	115.0	−0.294	0.0247	−0.940	0.0366
MMAB	122.9	0.260	0.0274	0.914	0.0327
ALDH7A1	3579.9	0.245	0.0291	0.924	0.0342
FDFT1	904.1	0.368	0.0369	0.800	0.0174
TK2	373.2	−0.196	0.0395	−0.807	0.0183
LOC107053589	196.3	0.405	0.0488	0.671	0.0049

1 Sample number: *N* = 6; OAZ1: ornithine decarboxylase antizyme 1; GPC2: None; FDPS: farnesyl diphosphate synthase; RGCC: regulator of cell cycle; PARP3: poly(ADP-ribose) polymerase family member 3; HSPB2: heat shock protein family B (small) member 2; DHCR24: 24-dehydrocholesterol reductase; MMAB: metabolism of cobalamin associated B; ALDH7A1: aldehyde dehydrogenase 7 family member A1; FDFT1: farnesyl-diphosphate farnesyltransferase 1; TK2: thymidine kinase 2.

### *GO and* KEGG pathway enrichment *analysis*

**Fig. 5:**
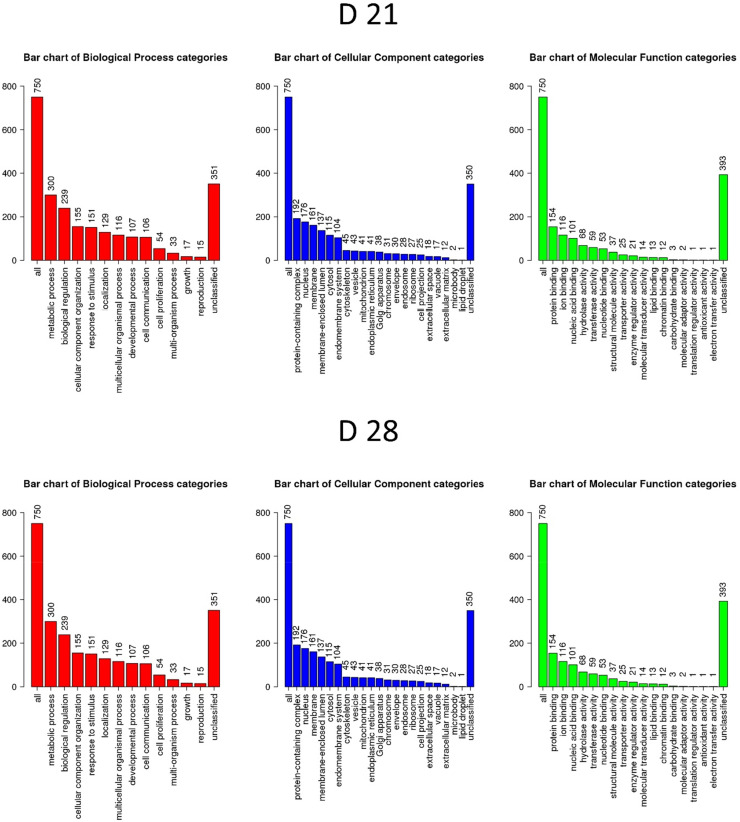
Gene ontology (GO) enrichment analysis for differentially expressed genes (DEGs) (*P* < 0.05) in the caudal area compared with the cranial area in chicken breast muscle on D 21 and D 28. Sample number: *N* = 6.

The results of the GO and KEGG pathway enrichment analyses using DEGs on D 21 and D 28 (*P* < 0.05) are shown in Fig. 5 and Table 4, respectively. Diverse GO terms in the biological process, cellular components, and molecular function categories were selected (Fig. 5), and these results suggested that there could be differences in muscle structures, protein synthesis, cellular pathways, nutrients, etc. between the caudal and cranial areas of chicken breast muscle. The KEGG pathway enrichment analysis indicated that various pathways, including regulation of actin cytoskeleton, ribosome, and focal adhesion, were regulated in the different areas of the breast muscle. DEGs related to lysine degradation according to KEGG pathway enrichment analysis on D 21 and D 28 are shown in Table 5. Lysine degradation is an important pathway in the mitochondria, so it could be associated with functions of mitochondria (Leandro and Houten, 2020). The modulation of mitochondria metabolism could be associated with breast myopathies (Shakeri et al., 2024; Zhang et al., 2024). The upregulation of genes associated with lysine degradation in the caudal area compared to the cranial area on D 21 contrasts to their downregulation on D 28. These findings suggest age-dependent variations in lysine degradation pathways between the caudal and cranial areas of breast muscle.

**Table 4:** Kyoto encyclopedia of genes and genomes (KEGG) pathway enrichment analysis by using differentially expressed genes (DEGs; *P* < 0.05) in the caudal area compared with the cranial area in chicken breast muscle on D 21 and D 281.

Gene Set	Description	Size	Significant compounds	*P* value
D 21				
gga00310	Lysine degradation	53	9	0.0004
gga04810	Regulation of actin cytoskeleton	187	19	0.0004
gga03010	Ribosome	119	15	0.0005
gga04520	Adherens junction	69	10	0.0007
gga04144	Endocytosis	224	20	0.0014
gga04012	ErbB signaling pathway	78	9	0.0059
gga04510	Focal adhesion	188	16	0.0066
gga00340	Histidine metabolism	21	4	0.0113
gga03013	RNA transport	133	12	0.0115
gga04910	Insulin signaling pathway	120	11	0.0137
D 28				
gga03010	Ribosome	119	31	<0.0001
gga03013	RNA transport	133	19	0.0004
gga03018	RNA degradation	69	19	0.0082
gga00310	Lysine degradation	53	7	0.0396

1 Sample number: *N* = 6.

**Table 5:** Differentially expressed genes (DEGs; *P* < 0.05) related to lysine degradation according to the Kyoto Encyclopedia of Genes and Genomes (KEGG) pathway enrichment analysis in the comparison of D 21 and D 281.

Gene name	Log_2_(fold change)	*P* value
D 21		
ALDH7A1	0.2448	0.02909
DOT1L	−0.3690	0.01034
KMT2A	−0.2888	0.00047
KMT2D	−0.7186	0.00001
NSD1	−0.2427	0.00484
PLOD1	−0.3309	0.01446
SETD2	−0.3786	0.00177
SETD3	0.2011	0.04835
SETDB1	−0.2407	0.01928
D 28		
ALDH7A1	0.2377	0.03423
DOT1L	0.2912	0.04256
EHHADH	0.4338	0.01374
GCDH	0.4462	0.00374
KMT2C	0.2626	0.03962
KMT2D	0.3833	0.01652
SETDB2	0.2925	0.01247

1 Sample number: *N* = 6; ALDH7A1: aldehyde dehydrogenase 7 family member A1; DOT1L: DOT1 like histone lysine methyltransferase; KMT2A: lysine methyltransferase 2A; KMT2D: lysine methyltransferase 2D; NSD1: nuclear receptor binding SET domain protein 1; PLOD1: procollagen-lysine, 2-oxoglutarate 5-dioxygenase 1; SETD2: SET domain containing 2, histone lysine methyltransferase; SETD3: SET domain containing 3, actin histidine methyltransferase; SETDB1: SET domain bifurcated histone lysine methyltransferase 1; EHHADH: enoyl-CoA hydratase and 3-hydroxyacyl CoA dehydrogenase; GCDH: glutaryl-CoA dehydrogenase; KMT2C: lysine methyltransferase 2C; SETDB2: SET domain bifurcated 2.

In summary, multivariate analyses showed that the gene expression profiles of the caudal and cranial areas in chicken breast muscle were not distinctly separated. The cranial area exhibited greater expression of genes associated with muscle synthesis and contraction, connective tissue, vascular health, and oxidative stress compared to the caudal area. These findings highlight significant regional differences within the muscle. Spatial variations in gene expression may be linked to functional or developmental differences between regions, potentially influencing muscle growth, meat quality, and breast myopathies. Understanding these spatial transcriptomic differences may provide additional insights into identifying etiologies and developing strategies for breast myopathies.

## Declaration of competing interest

The authors declare that they have no known competing financial interests or personal relationships that could have appeared to influence the work reported in this paper.

## References

[bib0001] Ayansola H., Liao C., Dong Y., Yu X., Zhang B., Wang B. (2021). Prospect of early vascular tone and satellite cell modulations on white striping muscle myopathy. Poult. Sci..

[bib0002] Baldi G., Soglia F., Mazzoni M., Sirri F., Canonico L., Babini E., Laghi L., Cavani C., Petracci M. (2018). Implications of white striping and spaghetti meat abnormalities on meat quality and histological features in broilers. Animal.

[bib0003] Barbut S.Y. (2025). Proc. Proceedings of the Australian Poultry Science Symposium.

[bib0004] Bian T., Xing T., Zhao X., Xu X. (2024). Effects of wooden breast myopathy on meat quality characteristics of broiler pectoralis major muscle and its changes with intramuscular connective tissue. Foods.

[bib0005] Brothers B., Zhuo Z., Papah M.B., Abasht B. (2019). RNA-seq analysis reveals spatial and sex differences in pectoralis major muscle of broiler chickens contributing to difference in susceptibility to wooden breast disease. Front. Physiol..

[bib0006] Che S., Wang C., Iverson M., Varga C., Barbut S., Bienzle D., Susta L. (2022). Characteristics of broiler chicken breast myopathies (spaghetti meat, woody breast, white striping) in Ontario. Canada. Poult. Sci..

[bib0007] Che S., Wang C., Varga C., Barbut S., Susta L. (2022). Prevalence of breast muscle myopathies (spaghetti meat, woody breast, white striping) and associated risk factors in broiler chickens from Ontario Canada. PloS One.

[bib0008] Choi J., Kong B., Bowker B.C., Zhuang H., Kim W.K. (2023). Nutritional strategies to improve meat quality and composition in the challenging conditions of broiler production: a review. Animals.

[bib0009] Choi J., Shakeri M., Kim W.K., Kong B., Bowker B., Zhuang H. (2024). Water properties in intact wooden breast fillets during refrigerated storage. Poult. Sci..

[bib0010] Clark D., Velleman S. (2016). Spatial influence on breast muscle morphological structure, myofiber size, and gene expression associated with the wooden breast myopathy in broilers. Poult. Sci..

[bib0011] CobbVantress. 2022. Cobb 500 broiler performance and nutrition supplement.

[bib0012] Collins K., Kiepper B., Ritz C., McLendon B., Wilson J. (2014). Growth, livability, feed consumption, and carcass composition of the Athens Canadian Random Bred 1955 meat-type chicken versus the 2012 high-yielding Cobb 500 broiler. Poult. Sci..

[bib0013] El-Mezgueldi M. (2014). Tropomyosin dynamics. J. Muscle Res. Cell Motil..

[bib0014] Galant C., Marchandise J., Stoenoiu M.S., Ducreux J., De Groof A., Pirenne S., Van den Eynde B., Houssiau F.A., Lauwerys B.R. (2019). Overexpression of ubiquitin-specific peptidase 15 in systemic sclerosis fibroblasts increases response to transforming growth factor β. Rheumatology.

[bib0015] Jiang X., Wu M., Xu X., Zhang L., Huang Y., Xu Z., He K., Wang H., Wang H., Teng L. (2019). COL12A1, a novel potential prognostic factor and therapeutic target in gastric cancer. Mol. Med. Rep..

[bib0016] Kalamajski S., Bihan D., Bonna A., Rubin K., Farndale R.W. (2016). Fibromodulin interacts with collagen cross-linking sites and activates lysyl oxidase. J. Biol. Chem..

[bib0017] Kanehisa M., Goto S. (2000). KEGG: kyoto encyclopedia of genes and genomes. Nucleic Acids Res..

[bib0018] Khatri B., Seo D., Shouse S., Pan J.H., Hudson N.J., Kim J.K., Bottje W., Kong B.C. (2018). MicroRNA profiling associated with muscle growth in modern broilers compared to an unselected chicken breed. BMC Genom..

[bib0019] Kong B.W., Hudson N., Seo D., Lee S., Khatri B., Lassiter K., Cook D., Piekarski A., Dridi S., Anthony N. (2017). RNA sequencing for global gene expression associated with muscle growth in a single male modern broiler line compared to a foundational Barred Plymouth Rock chicken line. BMC Genom..

[bib0020] Kong B., Owens C., Bottje W., Shakeri M., Choi J., Zhuang H., Bowker B. (2024). Proteomic analyses on chicken breast meat with white striping myopathy. Poult. Sci..

[bib0021] Kuttappan V., Hargis B., Owens C. (2016). White striping and woody breast myopathies in the modern poultry industry: a review. Poult. Sci..

[bib0022] Leandro J., Houten S.M. (2020). The lysine degradation pathway: subcellular compartmentalization and enzyme deficiencies. Mol. Genet. Metab..

[bib0023] Li D., Hou T., Du X., Zhao L., Zhang L., Gao F., Xing T. (2025). Integrated analysis of miRNA and mRNA expression profiles associated with wooden breast myopathy in broiler chickens. Int. J. Biol. Macromol..

[bib0024] Lin D., Alberton P., Caceres M.D., Volkmer E., Schieker M., Docheva D. (2017). Tenomodulin is essential for prevention of adipocyte accumulation and fibrovascular scar formation during early tendon healing. Cell Death Dis..

[bib0025] Moeller C., Swindell E.C., Kispert A., Eichele G. (2003). Carboxypeptidase Z (CPZ) modulates wnt signaling and regulates the development of skeletal elements in the chicken. Development.

[bib0026] Nallanthighal S., Heiserman J.P., Cheon D.J. (2021). Collagen type XI alpha 1 (COL11A1): a novel biomarker and a key player in cancer. Cancers.

[bib0027] Nishimura S., Ohtani M., Kabunda G.M., Arai S., Nishimura H., Hosaka Y.Z. (2024). Sex differences in COL1A1 expression and collagen content in skeletal muscle of mature and juvenile shamo chickens. J. Poult. Sci..

[bib0028] Papah M.B., Brannick E..M., Schmidt C.J., Abasht B. (2018). Gene expression profiling of the early pathogenesis of wooden breast disease in commercial broiler chickens using RNA-sequencing. PloS One.

[bib0029] Puolanne T.E.J., Costandache C.G., Ertbjerg P. (2021). Influence of woody breast myopathy on sarcomere length and tensile strength in commercial broiler pectoralis major muscle. Meat Muscle Biol..

[bib0030] Räisänen S.R., Lehenkari P.., Tasanen M., Rahkila P., Härkönen P.L., Väänänen H.K. (1999). Carbonic anhydrase III protects cells from hydrogen peroxide-induced apoptosis. The FASEB J..

[bib0031] Sand J., Genovese F., Karsdal M. (2016).

[bib0032] Scheuermann G., Bilgili S., Hess J., Mulvaney D. (2003). Breast muscle development in commercial broiler chickens. Poult. Sci..

[bib0033] Shahbandeh, M. 2023. Poultry industry in the United States - statistics & facts. https://www.statista.com/topics/6263/poultry-industry-in-the-united-states/#topicOverview. Accessed December 18 2023.

[bib0034] Shakeri M., Choi J., Harris C., Buhr R.J., Kong B., Zhuang H., Bowker B. (2024). Reduced ribonucleotide reductase RRM2 subunit expression increases DNA damage and mitochondria dysfunction in woody breast chickens. Am. J. Vet. Res..

[bib0035] Smith D., Fletcher D. (1988). Chicken breast muscle fiber type and diameter as influenced by age and intramuscular location. Poult. Sci..

[bib0036] Steppan J., Wang H., Bergman Y., Rauer M.J., Tan S., Jandu S., Nandakumar K., Barreto-Ortiz S., Cole R.N., Boronina T.N. (2019). Lysyl oxidase-like 2 depletion is protective in age-associated vascular stiffening. Am. J. Physiol. Heart Circ. Physiol..

[bib0037] Tasoniero G., Zhuang H., Bowker B. (2022). Biochemical and physicochemical changes in spaghetti meat during refrigerated storage of chicken breast. Front. Physiol..

[bib0038] Velleman S.G. (2020). Pectoralis major (breast) muscle extracellular matrix fibrillar collagen modifications associated with the wooden breast fibrotic myopathy in broilers. Front. Physiol..

[bib0039] Wang K., Meng X., Guo Z. (2021). Elastin structure, synthesis, regulatory mechanism and relationship with cardiovascular diseases. Front. Cell Dev. Biol..

[bib0040] Wells L., Edwards K.A., Bernstein S.I. (1996). Myosin heavy chain isoforms regulate muscle function but not myofibril assembly. EMBO J..

[bib0041] Wu Y., Chang T., Huang Y., Huang H., Chou C. (2014). COL11A1 promotes tumor progression and predicts poor clinical outcome in ovarian cancer. Oncogene.

[bib0042] Zhang X., Xing T., Zhang L., Zhao L., Gao F. (2024). Hypoxia-mediated programmed cell death is involved in the formation of wooden breast in broilers. J. Anim. Sci. Biotechnol..

[bib0043] Zhang Y., Huang M., Shao X., Zhang F., Li Z., Bai Y., Xu X., Wang P., Zhao T. (2023). Insights into intramuscular connective tissue associated with wooden breast myopathy in fast-growing broiler chickens. Foods.

[bib0044] Zhang Y., Park C., Bennett C., Thornton M., Kim D. (2021). Rapid and accurate alignment of nucleotide conversion sequencing reads with HISAT-3N. Genome Res..

[bib0045] Zhao F., Bai Y., Xiang X., Pang X. (2023). The role of fibromodulin in inflammatory responses and diseases associated with inflammation. Front. Immunol..

[bib0046] Zhu X., Puolanne E., Ertbjerg P. (2023). Changes of raw texture, intramuscular connective tissue properties and collagen profiles in broiler wooden breast during early storage. Foods.

[bib0047] Zou Y., Zwolanek D., Izu Y., Gandhy S., Schreiber G., Brockmann K., Devoto M., Tian Z., Hu Y., Veit G. (2014). Recessive and dominant mutations in COL12A1 cause a novel EDS/myopathy overlap syndrome in humans and mice. Hum. Mol. Genet..

